# Cell Fusion Reprogramming Leads to a Specific Hepatic Expression Pattern during Mouse Bone Marrow Derived Hepatocyte Formation *In Vivo*

**DOI:** 10.1371/journal.pone.0033945

**Published:** 2012-03-23

**Authors:** Oscar Quintana-Bustamante, Esther Grueso, Ramon Garcia-Escudero, Elvira Arza, Alberto Alvarez-Barrientos, Isabel Fabregat, Maria Garcia-Bravo, Nestor W. Meza, Jose C. Segovia

**Affiliations:** 1 Differentiation and Cytometry Unit, Hematopoiesis and Gene Therapy Division, Centro de Investigaciones Energéticas, Medioambientales y Tecnológicas (CIEMAT) and Centro de Investigación Biomédica en Red de Enfermedades Raras (CIBER-ER), Madrid, Spain; 2 Division of Medical Biotechnology, Paul Ehrlich Institute, Langen, Germany; 3 Molecular Oncology Unit, Epithelial Biomedicine Division, Centro de Investigaciones Energéticas, Medioambientales y Tecnológicas (CIEMAT), Madrid, Spain; 4 Microscopy and Dynamic Imaging Unit, Centro Nacional de Investigaciones Cardiovasculares, Madrid, Spain; 5 Bioscience Applied Techniques Facility, University of Extremadura, Badajoz, Spain; 6 Biological Clues of the Invasive and Metastatic Phenotype Group, Bellvitge Biomedical Research Institute (IDIBELL), Barcelona, Spain; 7 School of Medicine of Táchira, Universidad de los Andes, San Cristobal, Venezuela; Wellcome Trust Centre for Stem Cell Research, United Kingdom

## Abstract

The fusion of bone marrow (BM) hematopoietic cells with hepatocytes to generate BM derived hepatocytes (BMDH) is a natural process, which is enhanced in damaged tissues. However, the reprogramming needed to generate BMDH and the identity of the resultant cells is essentially unknown. In a mouse model of chronic liver damage, here we identify a modification in the chromatin structure of the hematopoietic nucleus during BMDH formation, accompanied by the loss of the key hematopoietic transcription factor PU.1/Sfpi1 (SFFV proviral integration 1) and gain of the key hepatic transcriptional regulator HNF-1A homeobox A (HNF-1A/Hnf1a). Through genome-wide expression analysis of laser captured BMDH, a differential gene expression pattern was detected and the chromatin changes observed were confirmed at the level of chromatin regulator genes. Similarly, Tranforming Growth Factor-β1 (TGF-β_1_) and neurotransmitter (e.g. Prostaglandin E Receptor 4 [Ptger4]) pathway genes were over-expressed. In summary, *in vivo* BMDH generation is a process in which the hematopoietic cell nucleus changes its identity and acquires hepatic features. These BMDHs have their own cell identity characterized by an expression pattern different from hematopoietic cells or hepatocytes. The role of these BMDHs in the liver requires further investigation.

## Introduction

For decades, the transformation of a given lineage of a cell into a completely different one has been suggested as the solution for numerous tissue specific diseases [1], [2], [3]. Using different approaches, this cell lineage switch has been widely explored [4]. Heterokaryon generation by *in vitro* cell fusion can modify the fate of differentiated cells [5]. Thus, it has been possible to reprogram different cell types to skeletal muscle cells by their *in vitro* fusion with muscle cells [6], [7]. The fusion of human B-lymphocytes with mouse embryonic stem cells can confer the human cells a multipotent state [8]. Through somatic cell nuclear transfer, it has also been possible to change the lineage of a cell to an embryonic stem cell identity with the capacity to act as a true embryonic stem cell and generate a complete organism [5], [9]. These two different cell reprogramming approaches indicate that all the elements and pathways required for the conversion of one cell type into another are present in cells. With this idea in mind, researchers have identified a subset of genes sufficient to transform a given cell type into a completely different type. This is the case of induced pluripotent stem cells (iPSC cells). Thus, the induced expression of 4 or fewer transcription factors can reprogram somatic cells to a more primitive state, equivalent to an embryonic stem cell [10], [11], [12]. Moreover, through the introduction of tissue specific transcription factors it has been possible to reprogram cells directly to other adult cell types [13], [14], [15].

Cell reprogramming involves modifying the program that gives rise to the initial specific lineage through gene silencing of the original transcription profile and acquisition and/or activation of new pathways from the acquired cell fate [5], [16]. This process occurs in a sequential manner during heterokaryon formation [6], [7], [8], [17], [18] and somatic cell nuclear transfer [19], [20], [21] or reprogramming by transcription factors [14], [22], [23], [24]. Indeed, physiologic cell reprogramming also occurs as a sequential process involving an intermediate undifferentiated state [23]. Chromatin remodeling genes play an important role in lineage transformation. Several epigenetic mechanisms have also been identified as contributing to cell lineage switching [25], [26], [27], [28], [29]. Changes in nuclear morphology have also been described [5]. However, it remains unclear whether epigenetic processes drive cell reprogramming or are just the result of cell transformation induced by lineage specific genes.

Cell fusion is a natural *in vivo* phenomenon that is highly regulated and required for development and homeostasis [30] but also occurs in disease processes such as virus-induced fusion [31] or tumorigenesis [32]. In some instances, cell fusion occurs between similarly differentiated cells to acquire completely new functions, such as the formation of osteoclasts from macrophages [33]. *In vivo* cell fusion has also been proposed as a cell reprogramming mechanism [4], [34] responsible, for example, for the generation of functional non-hematopoietic bone marrow derived cells, including muscle fibers, neurons or hepatocytes [35], [36], [37]. Reports of the generation of bone marrow-derived hepatocytes (BMDH) have mainly described the fusion of a myeloid hematopoietic cell lineage with hepatocytes [38], [39], [40]. The existence of BMDH has been widely reported in different species [41], [42] including humans [43], and their incidence may vary from rare to representing 20 to 40% of all hepatocytes [43], [44]. The appearance of BMDH is clearly dependent on the existence of hepatic damage [45]. Previously, we achieved an increase in BMDH frequency by treatment with granulocyte colony stimulating factor (G-CSF) in a mouse model of carbon tetrachloride-induced chronic liver damage [46]. However, despite extensive research, the processes of generation of BMDH and their possible roles remain unclear.

We here report that during the generation of BMDH, cell reprogramming occurs as a series of events in which changes in both the transcription of specific genes and nuclear structure are coordinated. In addition, we identified a BMDH-specific gene expression pattern reflecting observed chromatin organization changes and pointing to the BMDHs as a new cell identity.

## Results

### *In vivo* cell fusion induces nuclear heterochromatin remodeling

**Figure 1:**
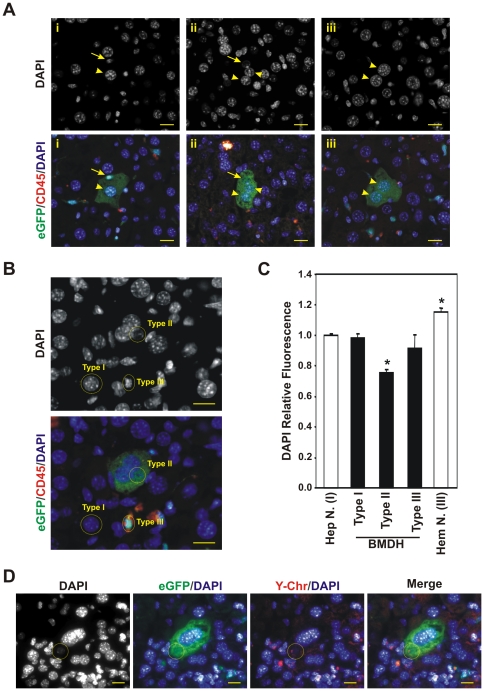
Nuclear chromatin structure modifications produced in the hematopoietic nucleus after *in vivo* cell fusion during BMDH formation. **A.** Presence of nuclei of different morphology in several BMDH as identified by immunofluorescence. Three examples (i, ii and iii) are shown. Arrowhead, BMDH nucleus with hepatocyte-like nuclear morphology; Arrow, BMDH nucleus with a different morphology to a hepatocyte nucleus. 20 µm scale bars are shown. **B.** Classification of the different BMDH nuclei according to their morphology and DAPI DNA-staining. Three different types of nuclei were defined; hepatocyte-like (Type I), hematopoietic-like (Type III) and non hepatocyte-non hematopoietic-like (Type II). 20 µm scale bars are shown. **C.** Histogram representing the quantification of DAPI relative fluorescence in the different BMDH (black bars), hepatocyte and hematopoietic cell nuclei (white bars). The data shown correspond to 57 nuclei of 35 BMDH examined in hepatic sections from several animals in two independent experiments ± SD. *p<0.05; **D.** Identifying the origin of BMDH nuclei by Y-FISH and immunofluorescence. Nuclei positive for the Y-chromosome arise from the exogenous BM. Yellow circle indicates one BM-derived nucleus. 20 µm scale bars are shown.

Heterokaryons are produced *in vivo* through cell fusion. It has been established that heterokaryon formation induces changes in the architecture of reprogrammed nucleus [7]. In this study, we investigated whether these chromatin changes take place during the formation of BMDH. To do this, a mouse model was used in which the hematopoietic system had been replaced with exogenous hematopoietic cells easily traceable. The female recipient mice were lethally irradiated and transplanted with whole Bone Marrow cells from male C57BL/6J-βactinEGFPxDBA/2 F1, which express the eGFP in around 80% of hepatocytes. Animals were subjected to chronic liver damage three months after bone marrow transplant for other three months followed by systemic treatment with G-CSF during three weeks, finally a month late the mice were sacrified. In prior work, we showed that this treatment significantly enhanced the appearance of BMDH [46]. BMDH were identified through their eGFP expression indicating a bone marrow origin, absence of the hematopoietic marker CD45, and hepatocyte morphology. The presence of different types of nuclei was observed in the BMDH which depended on cell size and DAPI staining pattern (Fig. 1A). Some BMDH had a single clear hepatocyte nucleus, which was large and had numerous nucleoli, but smaller nuclei could also exist that were brighter DAPI stained and resembled the nuclei of surrounding hematopoietic cells. Other BMDH showed nuclei that were of an intermediate size between a hematopoietic and hepatic nucleus; these were dimmer DAPI stained. Finally, we also identified BMDH whose nucleus or nuclei showed clear hepatocyte nucleus morphology.

To further explore the meaning of the different nuclear morphologies observed, we classified according to their nuclear morphology and DAPI staining a total of 57 nuclei of the 35 BMDH examined in four different hepatic sections per animal from a total of eight mice in two independent experiments. We established DAPI staining profiles for hepatocyte and hematopoietic nuclei in non fused cells (Figure 1B). The different BMDH nuclei were then classified depending on their size and DAPI staining pattern as: i) Type I, large nucleus of hepatic nuclear morphology, spherical, with many nucleoli (intensely DAPI stained); ii) Type II, medium-size nucleus, spherical, evenly stained, not bright and with few nucleoli; iii) Type III, small nucleus of hematopoietic nuclear morphology, not spherical, brightly stained, with few nucleoli (Figure 1B). Additionally, the DAPI staining pattern of each type of nuclei was maintained along the nucleus (Figures S4, S5, S6, S7); hematopoietic, hepatocyte and BMDH nuclei showed a homogeneous DAPI staining pattern in the different sections as analysed by confocal microscopy along the Z axis. Among 35 BMDH examined containing 57 nuclei (see Tables 1, 2 and 3), 18 had only one nucleus, 15 were binucleated and 2 contained more than two nuclei. The Type I nuclei were the most represented (around 50% of all the BMDH nuclei), followed by Type II (around 30%) and finally Type III (around 20%). Among the BMDH with a single nucleus, all three types defined were represented. Of the 35 BMDH analyzed, 11 BMDH had a Type I nucleus, 10 had a Type II nucleus and only 1 BMDH had a Type III nucleus. Additionally, we identified BMDH in which both Type I and Type II (4 BMDH out of 35) or Type III (9 BMDHs out of 35) nuclei coexisted, but no single BMDH showed the simultaneous presence of Type II and III nuclei.

**Table 1:** PU.1 Relative fluorescence (RF) of the three BMDH nucleus types defined.

	Type I nucleus						Type II nucleus				Type III nucleus	
BMDH	Nucleus A		Nucleus B		Nucleus C		Nucleus D		Nucleus E		Nucleus F	
	DAPI	RF	DAPI	RF	DAPI	RF	DAPI	RF	DAPI	RF	DAPI	RF
1	1.181	0.296									0.558	0.57
2	0.861	0.097									0.789	0.21
3	1.276	0.09	1.113	0.122								
4	1.214	0.035										
5											0.758	0.143
6	0.975	0.005										
7	0.841	0.066										
8	0.667	0.37									1.605	0.057
9	0.991	0.12									0.865	0.44
10							0.781	0.038				
11	0.67	0.198										
12							0.771	0.025	0.869	0.086		
13	1.098	0.024									1.174	1

Each row indicates the relative fluorescence (RF) values for DAPI and PU.1 (RF) of all nuclei detected in each BMDH were named alphabetically and classified according to its morphology (as Type I, II or III).

**Table 2:** HNF-1A Relative fluorescence (RF) of the three BMDH nucleus types defined.

	Type I nucleus						Type II nucleus				Type III nucleus	
BMDH	Nucleus A		Nucleus B		Nucleus C		Nucleus D		Nucleus E		Nucleus F	
	DAPI	RF	DAPI	RF	DAPI	RF	DAPI	RF	DAPI	RF	DAPI	RF
14	0.981	0.77									0.861	0.403
15	0.922	0.846										
16							0.852	0.888				
17							0.796	0.95				
18							1.006	1.394				
19	1.033	0.402	1.105	1.139								
20	0.89	0.521										
21	0.951	1.914										
22	0.912	1.211										
23	1.388	1.541									1.125	0.782
24							0.76	1.723				
25							0.633	1.06				
26							0.633	1				
27	1.012	1.123									0.705	0.877
28	1.01	0.559					0.707	0.966				
29	0.935	1.431	0.92	2.255								
39							0.663	0.348				
31			0.972	0.257			0.887	1.029				
32	1.204	1.245					0.752	0.714	0.545	0.245		
33	0.75	0.557	0.826	0.771	0.815	0.814	0.62	1.071				
34	0.954	0.541									0.836	0.492
35							0.708	0.945				

Each row indicates the relative fluorescence (RF) values for DAPI and HNF-1A (RF) of all nuclei detected in each BMDH were named alphabetically and classified according to its morphology (as Type I, II or III).

**Table 3:** Relative fluorescence (RF) of the three BMDH nucleus types defined.

	DAPI RF	PU.1 RF	HNF-1A RF
Hepatocyte nuclei	1.000±0.010 (n = 105)	0 (n = 39)	1 (n = 66)
Hematopoietic nuclei	1.156±0.022 (n = 105)	1 (n = 39)	0 (n = 66)
Type I nucleus	0.982±0.031 (n = 29)	0.129±0.035 (n = 11)	0.994±0.127 (n = 18)
Type II nucleus	0.751±0.028 (n = 17)	0.050±0.019 (n = 3)	0.940±0.106 (n = 14)
Type III nucleus	0.916±0.086 (n = 11)	0.403±0.143 (n = 6)	0.638±0.113 (n = 5)

Each row indicates the average of relative fluorescence (RF) values for DAPI and transcription factors (PU.1 RF and HNF-1a RF) in the nuclei of control cells (hematopoietic cells and hepatocytes).

Since the accessibility of DAPI to DNA depends on the extent of chromatin condensation [47], [48], we assessed chromatin condensation using a semi-quantitative approach based on DAPI staining intensity. The fluorescence densities of BMDH nuclei (*FD_DAPI_*) were calculated in relation to an average fluorescence intensity of normal hepatocytes (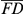 *_DAPINHep_*) to give a relative fluorescence value for each BMDH nucleus (Figure S1 and Tables 1, 2 and 3) as the ratio RF *(RF = FD*_DAPI_ */* 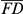
_DAPINHep_). These results are provided in Figure 1C. The RF of the hematopoietic cell nuclei was significantly higher than 1, indicating a greater extent of chromatin condensation than in the hepatocyte nuclei. In contrast, type I and type III BMDH nuclei showed a DAPI relative fluorescence close to 1. Most importantly, type II BMDH nuclei returned a RF value that was significantly lower than for the other types, indicating more chromatin condensation.

**Figure 2:**
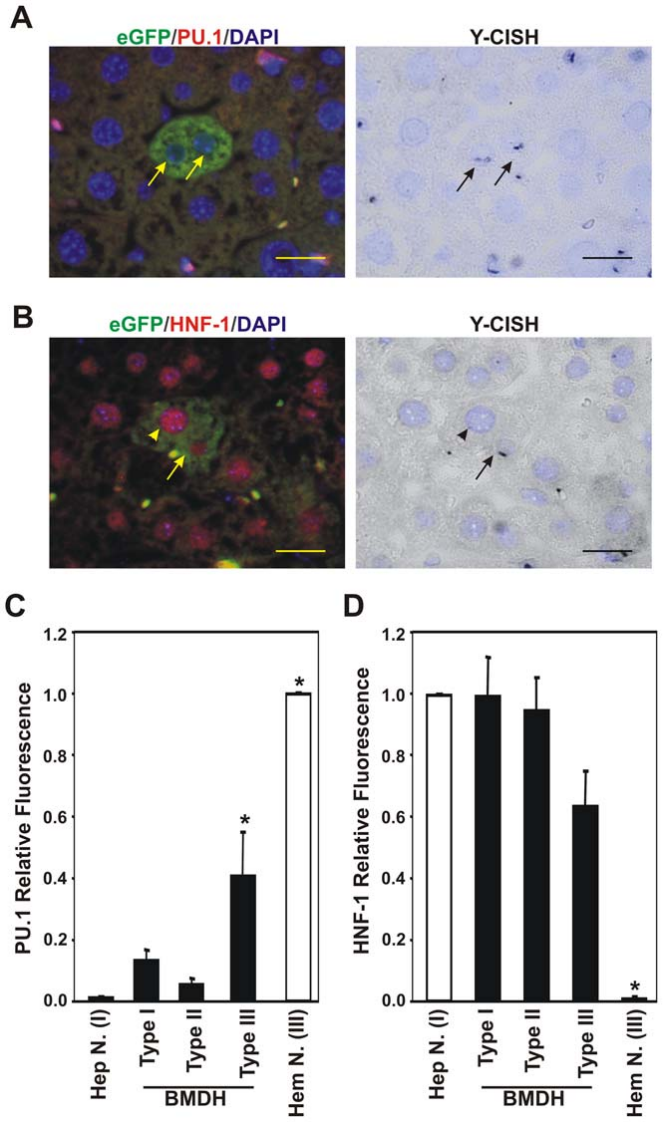
Gradual loss of master hematopoietic transcription factor PU.1 and gain of master hepatic transcription factor HNF-1A in BMDH nuclei. **A.** Absence of hematopoietic transcription factor PU.1 in two nuclei of a BMDH derived from BM cells (arrows) as identified by immunofluorescence and Y-CISH (black dots). 20 µm scale bars are shown. **B.** Presence of hepatic transcription factor HNF-1A in two nuclei of a BMDH derived from an endogenous hepatocyte (arrowhead) and a BM cell (arrow). 20 µm scale bars are shown. **C.** Quantification of PU.1 relative fluorescence in BMDH, hepatocyte and hematopoietic cell nuclei. Data shown correspond to 20 nuclei of 13 BMDH examined in hepatic sections from several animals in two independent experiments. Data expressed as means ± SD. *p<0.05. **D.** Quantification of HNF-1A relative fluorescence in BMDH, hepatocyte and hematopoietic cell nuclei. Data shown correspond to 37 nuclei of 22 BMDH examined in hepatic sections from several animals in two independent experiments. Data expressed as means ± SD. *p<0.05.

Additionally, we identified the origin of the different BMDH nuclei. Since besides expressing eGFP, cells derived from bone marrow contained the Y-chromosome (donor mice were male while recipients were female), we were able to identify the nuclei derived from the transplanted bone marrow by Y-FISH or Y-CISH (Figs. 1D, 2A, 2B and S2). Up to 24 nuclei were analyzed by Y-FISH. Half of them were endogenous and the other half were exogenous. All the endogenous nuclei have typical Type I morphology. However, the exogenous nuclei were of any morphology (2 Type I, 8 Type II and 2 Type III nuclei) (Figure S2B), indicating that nuclei from bone marrow cells could acquire the morphology of a hepatocyte nucleus.

Collectively these data suggest that after *in vivo* cell fusion, the nuclei of the fused hematopoietic cells undergo transformation to acquire a similar structure and chromatin condensation to those of a hepatocyte nucleus.

### Fused hematopoietic nuclei switch to a hepatic fate

To determine whether the transformation of fused hematopoietic nuclei was affecting their gene expression, we analyzed the expression of the key lineage-specific transcription factors of hematopoietic and hepatic programs, PU.1 and HNF-1A, respectively, by immunofluorescence. In addition, through simultaneous Y-CISH we identified the origin of the different BMDH nuclei. Most BMDH nuclei lacked PU.1 expression independently of their cell origin, only 5% of the BMDH nuclei maintained a PU.1 expression level similar to hematopoietic nuclei (Tables 1, 2 and 3). In contrast, all nuclei of infiltrated hematopoietic cells were positive for PU.1 (Fig. 2A). In BMDH, only type III nuclei (hematopoietic nucleus morphology) remained positive for PU.1 expression (Figure S2A). The loss of expression of this master hematopoietic gene was associated with the global hematopoietic expression program in the BMDH nuclei of hematopoietic origin, as revealed by the loss of the pan-hematopoietic marker CD45 (Fig. 1 and array data).

We then went on to address the acquisition of the hepatic program by detecting the hepatic transcription factor HNF-1A, essential for a hepatocyte identity. HNF-1A was detected in all the BMDH nuclei (Fig. 2B and Figure S2B). When we analyzed the presence of the Y-chromosome in the different BMDH nuclei, it was found that no BMDH nucleus of hematopoietic origin was negative for HNF-1A. These observations indicate that induction of the key hepatic transcription factor HNF-1A occurs soon after *in vivo* cell fusion and could activate the expression of hepatic or BMDH specific genes regulated by HNF-1A in all BMDH nuclei.

To explore the level of PU.1 and HNF-1A transcription factor expression in the BMDH nuclei, we calculated and normalized the presence of both PU.1 and HNF-1A transcription factors by quantifying their fluorescence intensity. In this semi-quantitative study, we estimated the fluorescence intensities of the specific hematopoietic and hepatic nuclear factors, PU.1 (*FI_PU.1_*) and HNF-1A (*FI_HNF-1A_*) respectively in BMDH nuclei relative to the fluorescence intensity of normal hematopoietic cells and hepatocytes present in the same hepatic tissue section (Figure S1). Next we calculated the relative fluorescence of PU.1 and HNF-1A for the different BMDH nuclei (Fig. 2C and 2D, Tables 1, 2 and 3). The nuclei of hematopoietic cells showed greatest PU.1 expression and this expression was reduced when these cells fused with hepatocytes. Interestingly, in type III nuclei, which are morphologically indistinguishable from hematopoietic cell nuclei, PU.1 expression was significantly higher than in the remaining BMDH or hepatocyte nuclei (Fig. 2C). In contrast, the presence of HNF-1A was highest in hepatocyte or type I and type II BMDH nuclei, and was reduced in type III nuclei (Fig. 2D).

All these observations suggest that when hematopoietic cells and hepatocytes fuse, the hematopoietic nuclei lose their expression pattern specific of its lineage and acquire hepatic key regulators. This is accompanied by a change in nuclear morphology, ending with the reprogramming of the hematopoietic identity of the BMDH nucleus to a hepatic fate.

### Gene expression profiling of BMDH reveals an intermediate state between hematopoietic and hepatic gene expression programs

**Figure 3:**
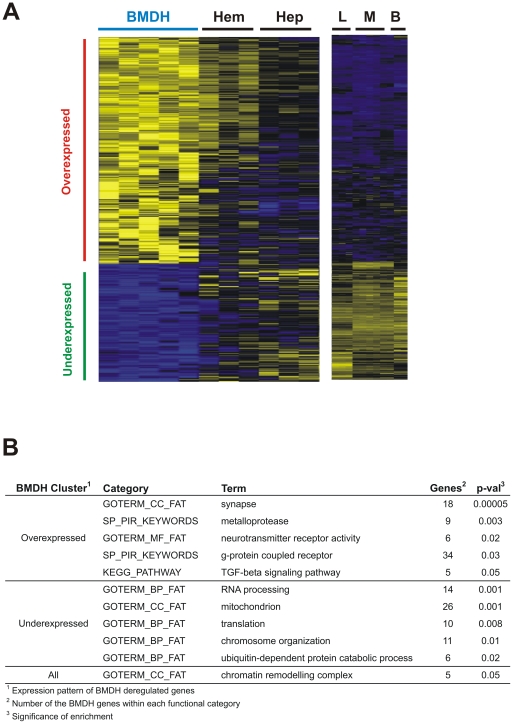
BMDH show a different gene expression profile to hematopoietic and hepatic cells. **A**. Heatmap of BMDH deregulated genes. Genes were selected based on Pearson's correlation on a template pattern of deregulated gene expression in BMDH cells, with respect to microdissected hepatocytes (Hep) and hematopoietic cells (Hem), and also to control mouse liver (L), macrophage (M) or B cells (B) (p-val<0.001) (see Materials and Methods). **B**. Functional annotation analysis of deregulated genes using the DAVID web tool (see Materials and Methods).

To further examine the reprogramming process observed in BMDH, we determined overall gene expression profiles in microdissected cells using Agilent Whole Mouse Genome Oligo Microarrays (see Materials and Methods and Figure S3). Microarray experiments were conducted on hepatic, hematopoietic and BMDH cells obtained from laser microdissected specimens (see Materials and Methods). Microdissected BMDH cells were not selected based on the type of nuclei, because RNA purification and SuperAmplification methodologies are not compatible with the staining process used to distinguish the different nuclei. Therefore, the profiling analysis has been designed to extract common patterns of gene expression in BMDH cells. Our findings identified 775 Agilent probes (corresponding to 561 genes) that were deregulated in BMDH with respect to microdissected hepatocytes and hematopoietic cells (Fig. 3A) (p-val<0.001) (see Materials and Methods). Underexpressed genes in BMDH (n = 207) were those involved in chromosome organization, RNA processing, translation, ubiquitin-dependent catabolism, and mitochondrial biology (Fig. 3B). In contrast, functional annotation analyses of the overexpressed genes in BMDH (n = 356) indicated roles in neurotransmission (including ligands or receptors), TGFβ signaling, and metalloprotease functions (Fig. 3B). The presence of genes associated with chromatin remodeling complexes was observed when both under and overexpressed genes where analyzed for enrichment (Fig. 3B), in line with the chromatin structural changes observed by DAPI staining (Fig. 1).

To identify the master regulators of the deregulated genes, we used the ChEA web tool (see Materials and Methods). ChEA contains a database of the results of published functional experiments on transcription factor binding in genomic DNA through chromatin immunoprecipitation. ChEA allows the identification through enrichment analysis of factors that act on a series of listed genes. Therefore, we decided to perform ChEA analysis using the lists of deregulated genes in BMDH, in order to extract potential transcription factors that would be regulating them, and therefore acting in the BMDH reprogramming process. Transcription factors participating in the regulation of embryonic development such as Myc, Pou5f1 (Oct4), Klf4, and Sox2, bind regulatory regions of overexpressed and underexpressed BMDH genes (Tables 4 and 5). Moreover, polycomb repressor complex (PRC) genes (Suz12, Eed, Rnf2 or Ring1b, and Ezh2) appeared to be modulating the transcription of overexpressed genes. Another transcription factor binding overexpressed BMDH genes is Jarid2 (Jumonji). This gene has been identified as essential for liver development and hepatocyte maturation [49].

**Table 4:** Transcription factor genes upregulated in BMDH.

Overexpressed		
TF-Expt ID^1^	Genes^2^	p-val^3^
EED-16625203	21	6.5E-05
EP300-20729851	49	8.2E-09
EZH2-18974828	40	1.4E-10
HNF4A-19822575	75	0.009
JARID2-20064375	34	4.8E-09
JARID2-20075857	37	2.4E-09
KLF4-18358816	29	0.002
KLF4-19030024	32	2.4E-05
MYC-18358816	42	0.05
MYC-19030024	49	0.02
MYC-19079543	22	0.02
MYC-19915707	50	6.9E-05
MYC-20876797	21	0.03
NANOG-16518401	48	0.007
NANOG-18347094	29	0.009
NANOG-18358816	21	0.008
NANOG-18692474	47	7.6E-04
NANOG-21062744	15	0.02
PAX3-FKHR-20663909	21	0.002
POU5F1-16518401	34	7.4E-06
POU5F1-18347094	30	0.02
POU5F1-18358816	13	0.03
POU5F1-18692474	73	3.7E-07
RCOR1-19997604	47	2.0E-06
REST-18959480	54	1.4E-06
RNF2-16625203	27	5.7E-05
RNF2-18974828	40	1.4E-10
SMAD1-18555785	15	9.4E-04
SOX2-18358816	14	0.02
SOX2-18555785	10	0.02
SOX2-18692474	53	1.4E-04
SOX2-19030024	20	3.0E-04
SOX2-21211035	60	3.0E-06
SUZ12-16625203	36	1.0E-08
SUZ12-18555785	31	5.5E-08
SUZ12-18692474	62	4.8E-17
SUZ12-18974828	58	1.6E-14
SUZ12-20075857	105	1.2E-19

ChEA software was used to identify transcription factors that could regulate the expression of BMDH genes with respect to microdissected cells (hematopoietic and hepatocytes) and mouse macrophages, B cells and liver tissue.

1 TF: transcription factor symbol; Expt ID: PubMed ID for the publication.

2 Number of the BMDH genes regulated by the TF.

3 Significance of the gene overlapping.

**Table 5:** Transcription factor genes downregulated in BMDH.

Underexpressed		
TF-Expt ID^1^	Genes^2^	p-val^3^
EP300-20729851	28	1.4E-04
EP300-21415370	13	0.01
HNF4A-19761587	26	1.7E-08
HNF4A-19822575	97	6.7E-20
KLF4-18358816	27	1.1E-05
KLF4-18555785	52	8.0E-15
KLF4-19030024	18	0.007
MYC-18358816	59	5.9E-13
MYC-18555785	29	8.7E-10
MYC-18940864	9	0.05
MYC-19030024	81	3.0E-23
MYC-19079543	28	2.0E-07
MYC-19915707	29	0.01
MYC-20876797	16	0.02
NANOG-16518401	34	0.008
NANOG-18347094	20	0.02
NANOG-18358816	17	0.002
NANOG-18555785	10	0.003
NANOG-18692474	50	3.4E-10
NANOG-21062744	12	0.007
POU5F1-18347094	35	1.7E-07
POU5F1-18358816	11	0.009
POU5F1-18555785	16	6.9E-07
POU5F1-18692474	66	1.7E-12
POU5F1-18700969	9	0.01
RCOR1-19997604	25	0.008
REST-18959480	29	0.008
REST-19997604	27	4.0E-04
SFPI1-20887958	44	1.1E-10
SMAD1-18555785	10	0.006
SMAD2-18955504	26	2.4E-04
SMAD3-18955504	26	2.4E-04
SMAD4-19686287	6	0.04
SOX2-18358816	10	0.03
SOX2-18555785	8	0.01
SOX2-18692474	55	2.2E-11
SOX2-19030024	17	4.1E-05
SOX2-20726797	30	7.7E-04
SOX2-21211035	43	8.8E-06

ChEA software was used to identify transcription factors that could regulate the expression of BMDH genes with respect to microdissected cells (hematopoietic and hepatocytes) and mouse macrophages, B cells and liver tissue.

1 TF: transcription factor symbol; Expt ID: PubMed ID for the publication.

2 Number of the BMDH genes regulated by the TF.

3 Significance of the gene overlapping.

In injured mouse liver, PU.1, which is expressed in most hematopoietic cells and whose expression is activated during myeloid and B-lymphoid cell development, showed reduced or no staining in BMDH cells (Fig. 2). Transcription factor enrichment analysis confirmed this pattern, since PU.1 targets were also down regulated in these cells (Table 5). We could not test whether HNF-1A was overexpressed in BMDH, as no ChIP experiment was included in the ChEA database. However, we did detect significant enrichment in genes regulated by HNF-4α (Table 4), which in turn regulates HNF-1A and may play a role in the differentiation of BM derived cells towards a hepatic fate.

Overall, ChEA enrichment indicated that BMDH are reprogrammed cells whose expression patterns are consistent with the gain of liver expression patterns at the expense of a loss in the typical expression profile of a hematopoietic lineage.

### BMDH acquire a specific gene expression fingerprint including deregulation of the TFGβ pathway and expression of neurotransmitters

**Figure 4:**
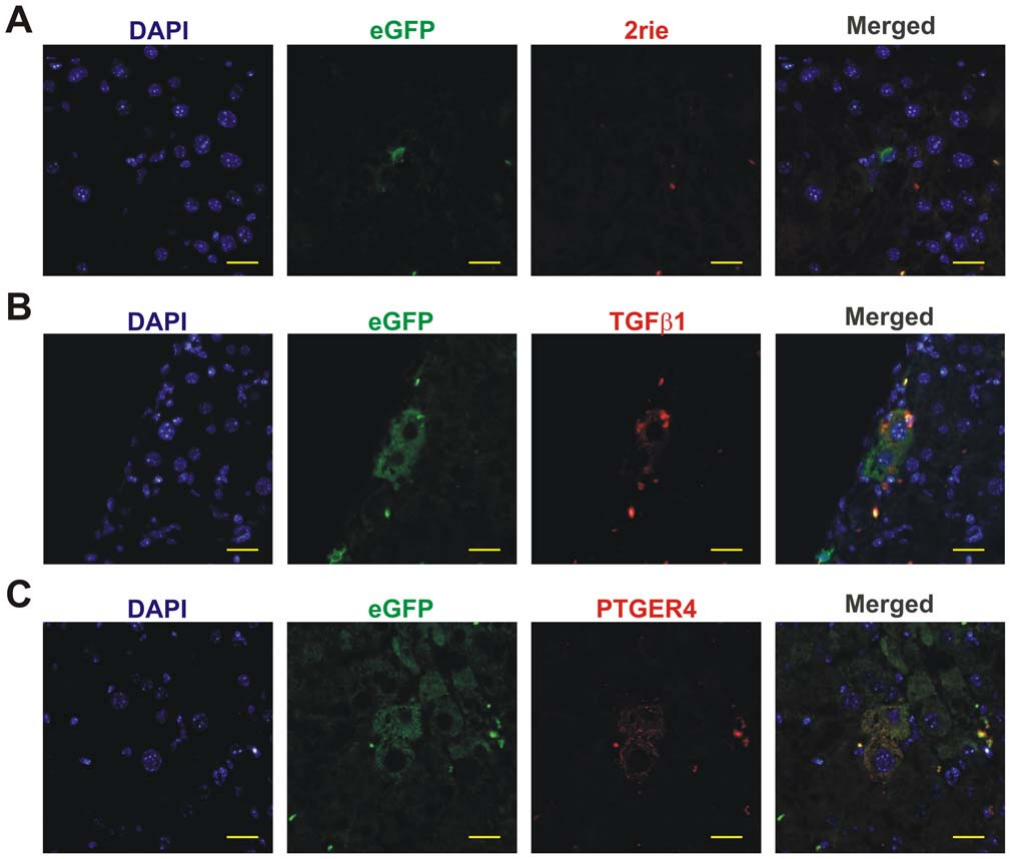
Tgfβ_1_ and Ptger4 are overexpressed in BMDH. **A.** Hepatic section sequentially stained, as described in Materials and Methods, for eGFP detection and with the secondary antibody donkey anti-rabbit-TexasRed. **B.** Tgfβ_1_ expression in BMDH. **C.** Ptger4 presence in BMDH. 20 µm scale bars are shown.

Interestingly, a considerable number of overexpressed genes observed in BMDH included those of the TGFβ pathway and neurotransmitters (Fig. 3B). This upregulated expression of Tgfβ_1_ and the neuroreceptor Ptger4 was confirmed by immunofluorescence labeling (Fig. 4). The presence of Tgfβ_1_ in BMDH (Fig. 4B) confirms the deregulation of TGFβ signaling during the reprogramming process. Ptger4 was also identified mainly in BMDH (Figure 4C), although a small proportion of other cells were also positive for this neuroreceptor (data not shown).

Functional annotation analyses revealed that several genes of the TGFβ signaling pathway, including Rps6kb1 (p70S6K), Nog (noggin), Inhbb (Inhibin β-B), Gdf5, and Smad9, are deregulated in BMDH cells. Additionally, TGFβ1 was detected in BMDH by immunofluorescence (Fig. 4B). Also, ChEA revealed that targets of Smad1, 2, 3 and 4 are downregulated, and that targets of Smad 1 and Pax3-Fkhr (involved in Smad2/3 signaling) are overexpressed (Tables 4 and 5). These results suggest that TGFβ pathway is deregulated during the reprogramming process. If this deregulation is a cause or effect in the BMDH generation is unknown.

Neurotransmitter genes found to be modulated in BMDH were those with a role in synaptic functions (Als2, Chrna5, Cplx2, Cplx4, Des, Dmxl2, Gabbr1, Gabra6, Gabrb1, Gabrg1, Glra2, Homer1, Itsn1, Myo7a, Otof, Phactr1, Rims1, Rps6kb1, Snph, Sv2b, Syn3, and Syt5) including neurotransmitters and neuroreceptors such as G-protein coupled receptors (GPCRs) (Table S1 and S2). Master regulators of neurogenesis are bound to the regulatory regions of both overexpressed and underexpressed genes, such as Rest or Rcor1 (Tables 4 and 5). Both these genes are transcriptional repressors that repress neuronal genes in non-neuronal tissues. As most synapse genes regulated by Rest or Rcor1 appeared overexpressed in BMDH, our data suggest that their repressor function was inhibited. The functional consequences of a neuronal program in BMDH cells remain to be determined.

## Discussion

*In vivo* fusion has been described as the main mechanism giving rise to non-hematopoietic bone marrow derived cells [35], [36], [37]. This process is promoted when there is tissue damage and the appearance of BMDH has even been associated with the amelioration of hepatic dysfunction [41]. Cell fusion is also thought to be a natural process that leads to the generation of tissues such as muscle fibers, and of specialized cells such as osteoclasts or giant cells from macrophages [30]. The cell reprogramming that takes place after cell fusion has been extensively explored *in vitro* [6], [7], [8] and despite BMDH having been widely reported [35], [36], [37], [40], [41], [45], [46], little is known about how similar processes occur *in vivo*. Moreover, a role for BMDH in hepatic repair has not been clearly established. In this study, we propose that the fusion of a hematopoietic cell with a hepatocyte triggers the reprogramming of the hematopoietic cell nucleus until it resembles a hepatocyte nucleus, both in its morphology and functionality. However, through a detailed gene expression analysis of these BMDH, we observed a distinct self-identity of these liver cells, as the overexpression of several components of the TGFβ pathway and of neurotransmission proteins involved in intercellular signaling.

In order to study BMDH generation, we have relied on eGFP expression in hepatocytes for identification of BMDH. We assumed a possible underestimation of 20% in the frequency of occurrence of BMDH, due to variegation in the expression of the marker transgen, where around 80% of hepatocytes from the transgenic mice used as BM donors, express eGFP. However, BMDH generation mechanism is independent of the expression of eGFP, as in transgenic mice themselves the expression of this marker gene does not affect any cell type. In our model, we have analysed a snapshot of this process where we have been able to identify differences in BMDHs, which might mean different stages in the *in vivo* cell fusion reprogramming process.Once fused with a hepatocyte, the hematopoietic cell nucleus starts its transformation. As other authors have described previously *in vitro* [5], [7], we have observed a similar process might take place in our *in* vivo model. First, by differential DAPI staining, we noticed a change in nuclear structure. In BMDH, the non-hepatic nucleus is enlarged while it becomes more inaccessible to the DNA intercalating agent DAPI (Fig. 1). The gradual reduction in fluorescence emitted by DAPI defines the transition from an early stage of the so-called type III nucleus, hematopoietic-like in shape, to an intermediate type II nucleus. We have been demonstrated the bone marrow origin of all the Type III and Type II nuclei, and also of some of Type I using Y-FISH, indicating their hematopoietic origin. This transition is also characterized by a gradual increase in hepatic transcription factor HNF-1A and the sudden non-expression of the hematopoietic transcription factor PU.1 in the changing nucleus. This nuclear transformation ends when the non-hepatic nucleus becomes a hepatocyte-like nucleus (type I nucleus), which is morphologically indistinguishable from an endogenous hepatocyte nucleus and only expresses hepatic transcription factor HNF-1A. Similar changes in nuclear structure, nuclear enlargement and chromatin modification, have been described during the generation of heterokaryons *in vitro* [5], [7]. These changes have been related to a process of chromatin remodeling [7], [17], [18], [25] involving mechanisms such as demethylation of tissue-specific gene promoters [8], [18] or histone deacetylation [7], [17], among others. Here we were able to track chromatin remodeling through the detection of nuclear reshaping and modifications deduced from the microarray analyses.

Our array data reveal the important role of chromatin modification in *in vivo* BMDH generation. For example, NuSAP plays a crucial role in spindle microtubule organization; it is expressed during the transition from G2 to mitosis and localized in the nucleoli during interphase [50]. Thus, the observed overexpression of NuSAP in BMDH could contribute to the formation of a hepatocyte-like nucleus with numerous nucleoli. In contrast, BMDHs show reduced expression of the genes SATB2, BCOR and Ep400. SATB2 regulates the expression of several genes (e.g. Nanog [51]) and the chromatin structure of multigene clusters (e.g. Hox gene clusters [52]). Its reduction in BMDH could cause gene silencing during cell reprogramming. BCOR binds to BCL6 to exert a repressive role in B cells and polycomb family members of the BCOR-BCL6 complex are also capable of gene silencing [53]. Ep400 forms part of a large chromatin-remodeling complex and is essential for the expression of Hox genes in hematopoiesis and for cell cycle progression [54], [55]. On the other hand, according to our ChEA analyses, polycomb repressor complex (PRC) genes (Suz12, Eed, Rnf2 or Ring1b, and Ezh2) appear to modulate the transcription of overexpressed genes. Since PRC genes are protein repressors, targeted proteins may detach from the promoters during BMDH reprogramming, allowing the induction of target genes. The alteration of these genes and others involved in nuclear structure remodeling could play an important role in the cell reprogramming of BMDH.

Here we clearly show a change in the expression of key tissue-specific genes in the nuclei of the fused hematopoietic cells. PU.1 disappears from the type III nuclei to the transition type II nuclei, but HNF-1A appears at an early stage in the new BMDH nuclei (Fig. 2). The reduction in levels of PU.1 indicates early silencing of the hematopoietic expression program of these nuclei (Tables S1 and S2). During the formation of heterokaryons between mouse muscle cells and human keratinocytes, cell reprogramming was recently described as a fast process [6]. This would explain why the type III nuclei present in a fused cell are HNF-1A positive, but have not yet fully silenced PU.1 (Fig. 2C and 2D).

It may be argued that the presence of HNF-1A in a type III nucleus is the outcome of activation of a hepatic program in this nucleus or may be due to migration of this transcription factor to the exogenous nucleus. Regardless of the source of HNF-1A, BMDH nuclei arising from hematopoietic cells became hepatic-like nuclei as demonstrated by the presence of the Y-chromosome (Fig. 2 and Figure S2). It has been broadly demonstrated that specific transcription factors can promote the generation of cells of a completely different fate [10], [12], [13], [14], [28], [34], [56], [57]. Thus, the hepatic transcription factors present in our heterokaryons could induce the reprogramming of the hematopoietic nucleus to a hepatic fate.

The cell reprogramming process may even be bidirectional [6]. In our model, the hematopoietic cell might reprogram the hepatocyte nucleus in the fused cells. We may assume that the balance here is shifted towards acquiring a hepatic phenotype for two main reasons: i) the direction of reprogramming by fusion *in vitro* depends on the ratio between the two cell types [6], and ii) extracellular signals in the liver are able to transdifferentiate hematopoietic cells to hepatocytes [58].

Using different techniques we found that the key hematopoietic transcription factor PU.1 was silenced in BMDH. Our ChEA analysis also revealed that some PU.1 gene targets are also deregulated in BMDH. Some of these PU.1 targets are involved in chromatin remodeling (Aebp2, Chd9, Rbbp7, or Tnks2), suggesting that PU.1 regulates reprogramming processes in BMDH cells. The lack of PU.1 expression could facilitate cell reprogramming via chromatin remodeling changes due to the down regulation of specific chromatin remodeling genes. In addition, the key hepatic transcription factors HNF-1A and HNF-4α were identified here in BMDH by immunofluorescence and microarrays analysis, respectively. Ectopic HNF-4α expression seems to be needed to direct *in vitro* hepatic differentiation from BM cells [57]. Whether HNF-1A is positively activated by HNF-4α in BMDH remains to be determined, but we suggest that a liver specific expression program associated with HNF factors comes into play.

In *Caenorhabditis elegans*, direct *in vivo* reprogramming generates undifferentiated intermediate states that precede redifferentiation into the new cell type [23]. Our array data indicate that the transcription pathways regulated by the pluripotent transcription factors Oct-4, Klf-4, Sox2, and also c-Myc, are clearly modulated in BMDH, suggesting the existence of an intermediate and undifferentiated state (i.e. type II nuclei).

According to our array data, the final conversion of BMDH into a hepatocyte is not complete. Thus, in our case the fused nucleus may preserve part of its hematopoietic identity and continue to express certain hematopoietic genes or hematopoietic epigenetic marks (Tables S1 and S2). As is likely for genes involved in chromatin organization, these genes specific to BMDH could participate in the process of cell reprogramming, or could represent the acquisition of a new role of newly generated BMDH. In effect, the genes showing clear differential expression in BMDH include those coding for various components of the TGFβ pathway, several neuroreceptors and cytokines (Fig. 3 and Tables S1 and S2). Among these, we show here by immunofluorescence the differential protein expression of TGFβ and PTGER4 in BMDH. The TGFβ pathway has been widely incriminated in liver repair processes [59]. BMDH are mainly generated in the context of hepatic damage [46] such that it is likely that these BMDH participate in the liver repair mechanism. Moreover, the genes specific to BMDH encoding neuroreceptors (Tar, Agtrl1, Npffr, Oxtr, Par, Ptger4, Trhr, Crhr, Grm, Gabra6, Gabrb1, Gabbr1 and Lepr) or cytokines (Ccl28, Il12b, Csf2, Ifna13, Inbb and Gdf5) take part in different signaling processes, pointing to a possible signaling role of BMDH in hepatic repair.

## Materials and Methods

### Animal procedures and tissue collection

All experimental procedures were approved by the ethics committee of the CIEMAT accorded to Spanish and European directives (Approval ID# 28079-21A of the Ministerio de Medio Ambiente, Medio Rural y Marino). C57BL/6JxDBA/2 F1 female mice were subjected to lethal irradiation and intravenously injected with 1·10^7^ whole bone marrow cells harvested from C57BL/6J-βactinEGFP (kindly provided by Dr. M. Okabe, Osaka, Japan)×DBA/2 F1 male mice. Three months post transplantation, the mice were intraperitoneally injected with 1·10^−2^ mol/Kg of body weight of CCl_4_ (Fluka, Buchs, Switzerland) in olive oil on a weekly basis for three months. To induce BMDH formation, prior to sacrifice the hepatic injured animals were hematologically mobilized through a 3-week course of subcutaneous injections of 50 µg of pegylated granulocyte colony stimulating factor (Neulasta, Amgen, Breda, Netherlands) in PBS/0.1% BSA.

For tissue collection, animals were transcardially perfused with 10 ml cold PBS/20 mM EDTA, followed by 25 ml cold 4% paraformaldehyde (PFA, Merck, Darmstadt, Germany). Liver lobes were fixed in 4% formalin and paraffin-embedded or incubated in 30% sucrose/PBS at 4°C overnight and kept in OCT (Sakura Finetek, Zoeterwoude, Netherlands) at −80°C until analysis.

### Immunohistological analysis

All immunofluorescence and *in situ* hybridization procedures were performed on 5-µm sections of paraffin-embedded tissue. For the co-localization of enhanced GFP (eGFP) and CD45, tissue sections were digested with Proteinase K (Dako, Carpinteria, CA), blocked and incubated with rabbit anti-eGFP (20 µg/mL, Molecular Probes, Eugen, OR) or goat anti-eGFP (Abcam, Cambridge, UK) and biotinylated anti-CD45 (1.25 µg/mL, 30-F11 clone, BD Bioscience Pharmingen, San Jose, CA) antibodies. After washing, the samples were incubated with donkey anti-rabbit AlexaFluor®488 or Donkey anti-goat-FITC (Jackson ImmunoReseach, West Grove, PA) and streptavidin AlexaFluor®594 antibodies (2 µg/mL and 4 µg/mL respectively, Molecular Probes). Finally, nuclei were stained with 4,6-diamidino-2-phenylindole (DAPI, Boehringer, Ingelheim, Germany) dissolved in Mowiol (Sigma-Aldrich, Steinheim, Germany) as mounting medium. For nuclear staining, a multistep protocol was performed. In brief, tissue sections were treated with boiling citrate buffer (10 mM sodium citrate/4.4 mM chloridric acid, pH 6), blocked, incubated with rabbit anti-PU.1 (8 µg/mL, T-21 clone, Santa Cruz Biotechnology, Santa Cruz, CA) rabbit anti-HNF-1A (8 µg/mL, H-205 clone, Santa Cruz Biotechnology), rabbit anti-PTGER4 (40 µg/mL, MBL, MA) or rabbit anti-TGFβ_1_ (Santa Cruz Biotechnology) antibodies overnight at 4°C. After washing and adding donkey anti-rabbitTexasRed (7.5 µg/mL, Jackson Immunoresearch Laboratories, Cambridgeshire, UK), the samples were washed, blocked, incubated sequentially with rabbit anti-eGFP and donkey anti-rabbit AlexaFluor®488, and mounted. The tissue sections were analysed with an Axioplan 2 imaging fluorescent microscope (Zeiss, Jena, Germany) or with Bio-Rad Radiance 2100 confocal system (Zeiss) when Z-stack analyses were performed.

### Fluorescence quantification

Four tissue sections from different hepatic lobes per animal of eight animals of two independent experiments were used for the fluorescence quantification of DAPI, PU.1 and HNF-1. To quantify the density of fluorescence due to DAPI staining of nuclei, we used Image Tool 3.00 software (University of Texas, San Antonio, TX). The fluorescence density of different nuclei was calculated in the blue channel; DAPI relative fluorescence (Figure S1) for each nucleus was defined as the relationship between the DAPI fluorescence density of a nucleus with respect to the arithmetic mean of the DAPI fluorescence density values obtained for hepatocyte nuclei used as controls in each image. To quantify relative fluorescence for PU.1, we determined the PU.1 fluorescence density value and PU.1 fluorescence density values for the nuclei of three different hematopoietic cells and hepatocytes; thereby avoiding differences in fluorescence between different images. DAPI relative fluorescence was calculated for 57 BMDH, 105 hepatocyte and 105 hematopoietic nuclei. We calculated PU.1 relative fluorescence for each BMDH nucleus and control nuclei according to the equation in Figure S1, in which PU.1 nuclear fluorescence is referred to the PU.1 fluorescence of hematopoietic cell nuclei after subtracting the autofluorescence of hepatocyte nuclei. Thus, PU.1 relative fluorescence of the hematopoietic nuclei will be 1 and that of the hepatocyte nuclei will be 0. PU.1 relative fluorescence was calculated for 20 BMDH, 39 hepatocyte and 39 hematopoietic nuclei. Similarly, we calculated HNF-1A relative fluorescence using the equation in Figure S1, in which HNF-1A nuclear fluorescence is referred to the HNF-1A fluorescence of hepatocyte nuclei once the autofluorescence of the hematopoietic cell nuclei in the red channel has been subtracted. This time the HNF-1A relative fluorescence of the hepatocyte nuclei will be 1 and that of hematopoietic cell nuclei will be 0. HNF-1A relative fluorescence was calculated for 37 BMDH, 66 hepatocyte and 66 hematopoietic nuclei. Relative fluorescence values were compared using the nonparametric Wilcoxon Mann-Whitney test and Kruskal Wallis test implemented in the Statgraphics software package (Manugistic Inc, Rockville, MD). Data are expressed as the mean ± standard error. The level of statistical significance was set at p<0.05.

### Y-chromosome *in situ* hybridization

To simultaneously detect the presence of the Y chromosome and eGFP expression, Y-chromosome fluorescence *in situ* hybridization (Y-FISH) was conducted using the Star FISH Kit (Cambio, Cambridge, UK) according to the manufacturer's instructions. On the same sections, eGFP expression was identified by immunofluorescence as described above. The presence of the Y chromosome in the BMDH previously stained for different nuclear factors was detected by Y-chromosome chromogenic *in situ* hybridization (Y-CISH) and developed using the ABC kit (Vector Laboratories, Burlingame, CA).

### Laser cell capture and mRNA superamplification

For laser capture of individual cells, 12–15 µm frozen sections were analyzed under an Olympus IX81 motorized inverted microscope equipped with a laser catapulting microdissection device (Zeiss, Jena, Germany) (Figure S3). BMDH were identified according to their hepatocyte morphology, eGFP expression and lack of autofluorescence; hepatocytes according to their morphology and lack of eGFP expression; and hematopoietic cells according to their smaller size and eGFP expression. Tissue surrounding the cells of interest was destroyed by laser-burning. These isolated cells were catapulted to Eppendorf caps and then lysed in SuperAmp lysis buffer and stored at −80°C according to the instructions of the SuperAmp Preparation kit (Miltenyi Biotec, Bergisch Gladbach, Germany). SuperAmplification was performed according to Miltenyi Biotec's undisclosed protocol. The integrity of cDNA was checked using the Agilent 2100 Bioanalyzer platform (Agilent Technologies, Palo Alto, CA). The average length of the library PCR products was 200–1000 bp.

### Gene expression microarray analysis

Superamplified cDNA was hybridized according to the Agilent 60-mer oligo microarray processing protocol employing the Agilent Gene Expression Hybridization kit (Agilent Technologies) utilizing 1.25 µg Cy3-labeled fragmented cRNA on Agilent whole mouse genome oligo microarrays. As controls we used microarray data obtained using the same Agilent chip for mouse macrophages, B cells and liver tissue [60]. Raw data were downloaded from the GEO database (datasets GSE21512 and GSE14921). The Agilent feature extraction software (FES) was used to read out and process the microarray image files. Raw data were normalized by quantile normalization [61]. Two BMDH samples were discarded because of defective normalization. The final dataset contained the following biological replicates: 5× BMDH, 3× hepatocytes, and 3× hematopoietic cells. Genes deregulated in BMDH cells were extracted by Pavlidis template matching [62]. Briefly, Pearson's correlation coefficient is computed between the intensities measured for each gene and the values of an independent variable. P-values to test for the null hypothesis that the correlation is zero are calculated. The independent value acts as a template where certain expression patterns could be analyzed. The independent value (template) was selected to search for genes overexpressed (or underexpressed) in BMDH cells with respect to microdissected cells (hematopoietic and hepatocytes) and mouse macrophages, B cells and liver tissue. The threshold significance values used were: p-val<0.001 and Pearson coefficient R>0.65 (overexpressed in BMDH) or R<−0.65 (underexpressed in BMDH). Enrichment analyses of Gene Ontology terms and KEGG pathways were performed using the web utility DAVID (http://david.abcc.ncifcrf.gov) [63]. ChIP Enrichment Analysis (ChEA) software (http://amp.pharm.mssm.edu/lib/chea.jsp) was used to search for transcription factors that could be controlling the expression of the genes deregulated in BMDH cells [64]. Briefly, a database of ChIP-chip, ChIP-seq, ChIP-PET and DamID experiments, whereby interactions of specific transcription factors with their DNA binding sites are determined, was generated (ChIP-X database) [64]. The database contains 189,933 interactions, manually extracted from 87 publications, describing the binding of 92 transcription factors to 31,932 target genes. We used this database to analyze BMDH mRNA expression data. ChEA software computes over-representation of transcription factor targets from the ChIP-X database. The output of the analysis is the factors that could be acting in the BMDH cells, and the number and list of BMDH deregulated genes regulated by each factor. Same transcription factor could appear more than once (such as MYC, NANOG, etc), as different experiments have been reported for the same factor (identified by the PMID of each report). Raw and processed microarray data generated in this study accomplish MIAME guidelines, and has been deposited in the GEO database under the accession number GSE29878.

## Supporting Information

Figure S1 **Mathematical equations used to calculate relative fluorescence (RF) values for DAPI, PU.1 and HNF-1A (see Experimental Procedures).**
(TIF)

Figure S2 **Identification of PU.1 and HNF-1A in BMDH nuclei.** Additional examples of identification of PU.1 and HNF-1A in BMDH by immunofluorescence and Y-CISH. **A.** PU.1 analysis in the nuclei of BMDH originating from endogenous hepatocytes (arrowhead) or BM cells (arrows). **B.** Presence of HNF-1A in a multinucleated BMDH originating from endogenous hepatocytes (arrowhead) or BM cells (arrows). 20 µm scale bars are shown.
(TIF)

Figure S3 **Laser capture procedure used to obtain isolated BMDH for further molecular analyses.** BMDH were selected according to a clear hepatocyte like morphology (**A**) and their eGFP expression (**B**). **C.** To ensure that only the selected cell is captured, the tissue surrounding the cell of interest is burned out with the laser beam. **D.** The selected cell is catapulted to an Eppendorf tube cap for further RNA extraction.
(TIF)

Figure S4 **Z-stack confocal analysis.** DAPI staining pattern of different nuclei (**A**) and identification of eGFP (green), CD45 (red) and DAPI (blue) staining (**B**) along Z-axis is represented as a serial 0.25 µm frames separate each 1 µm. BMDH (dotted line), hematopoietic (arrowhead) and hepatocyte (arrow) nuclei are shown.
(TIF)

Figure S5 **Z-stack confocal analysis of a BMDH with a Type I nucleus.** DAPI staining pattern of different nuclei (**A**) and identification of eGFP (green), CD45 (red) and DAPI (blue) staining (**B**) along Z-axis is represented as a serial 0.25 µm frames separate each 1 µm. BMDH (dotted line) and hepatocyte (Type I, asterisk) nuclei are shown.
(TIF)

Figure S6 **Z-stack confocal analysis of a BMDH with a Type I and a Type II nuclei.** DAPI staining pattern of different nuclei (**A**) and identification of eGFP (green), CD45 (red) and DAPI (blue) staining (**B**) along Z-axis is represented as a serial 0.25 µm frames separate each 1 µm. BMDH (dotted line), Type II (arrowhead) and hepatocyte (Type I, asterisk) nuclei are shown.
(TIF)

Figure S7 **Z-stack confocal analysis of a BMDH with a Type I and a Type III nuclei.** DAPI staining pattern of different nuclei (**A**) and identification of eGFP (green), CD45 (red) and DAPI (blue) staining (**B**) along Z-axis is represented as a serial 0.25 µm frames separate each 1 µm. BMDH (dotted line), Type III nucleus (arrowhead) and hepatocyte nucleus (Type I, asterisk) are shown.
(TIF)

Table S1 **Overexpressed genes in BMDH.**
(XLS)

Table S2 **Underexpressed genes in BMDH.**
(XLS)
