# Supplementary figures and images for: Cell Fusion Reprogramming Leads to a Specific Hepatic Expression Pattern during Mouse Bone Marrow Derived Hepatocyte Formation In Vivo
Source: PLoS One. 2012 Mar 23;7(3):e33945. doi: 10.1371/journal.pone.0033945 (PMC3311566; doi:10.1371/journal.pone.0033945)

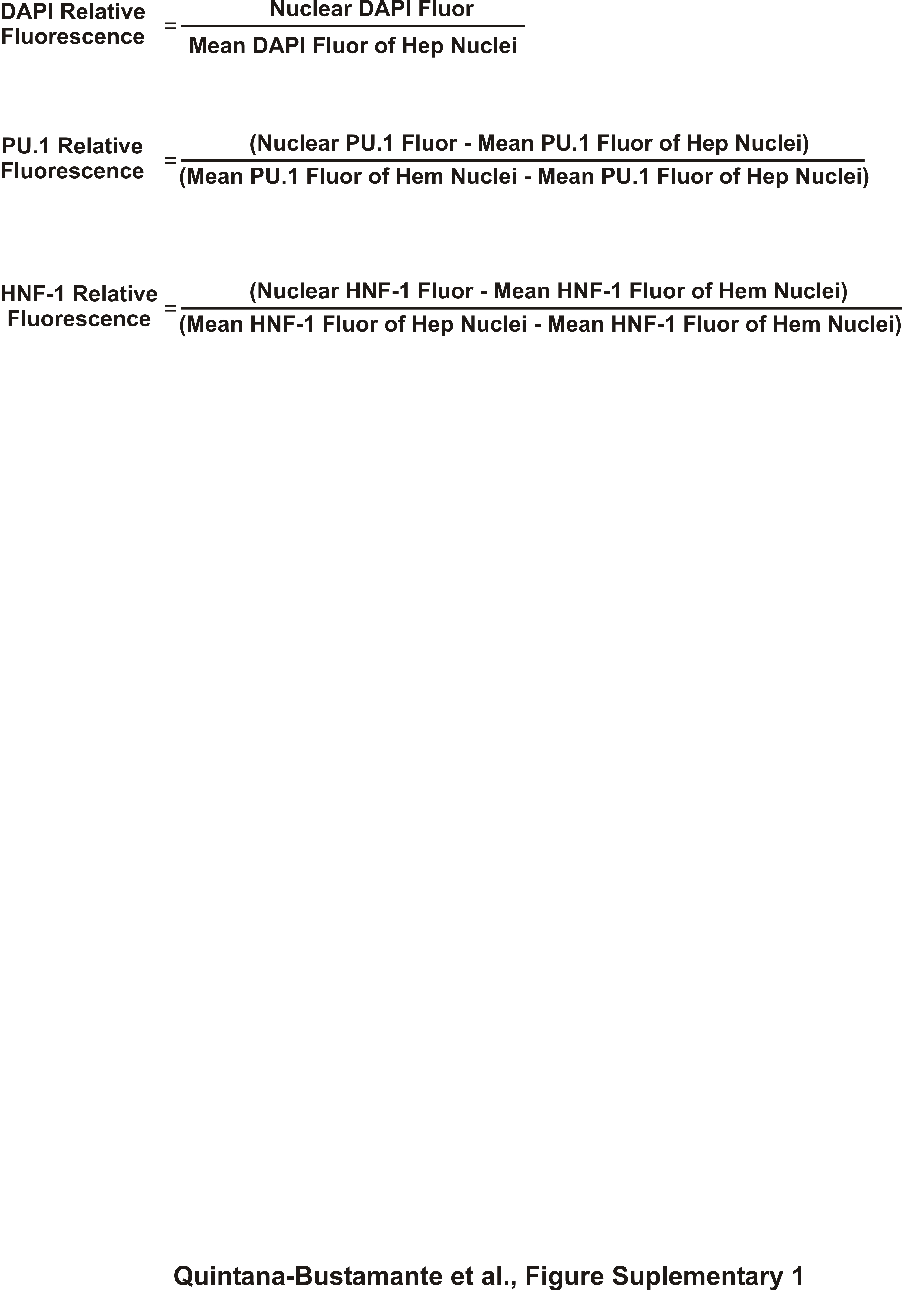

Supplement: Figure S1 — Mathematical equations used to calculate relative fluorescence (RF) values for DAPI, PU.1 and HNF-1A (see Experimental Procedures). (TIF) [file pone.0033945.s001.tif]

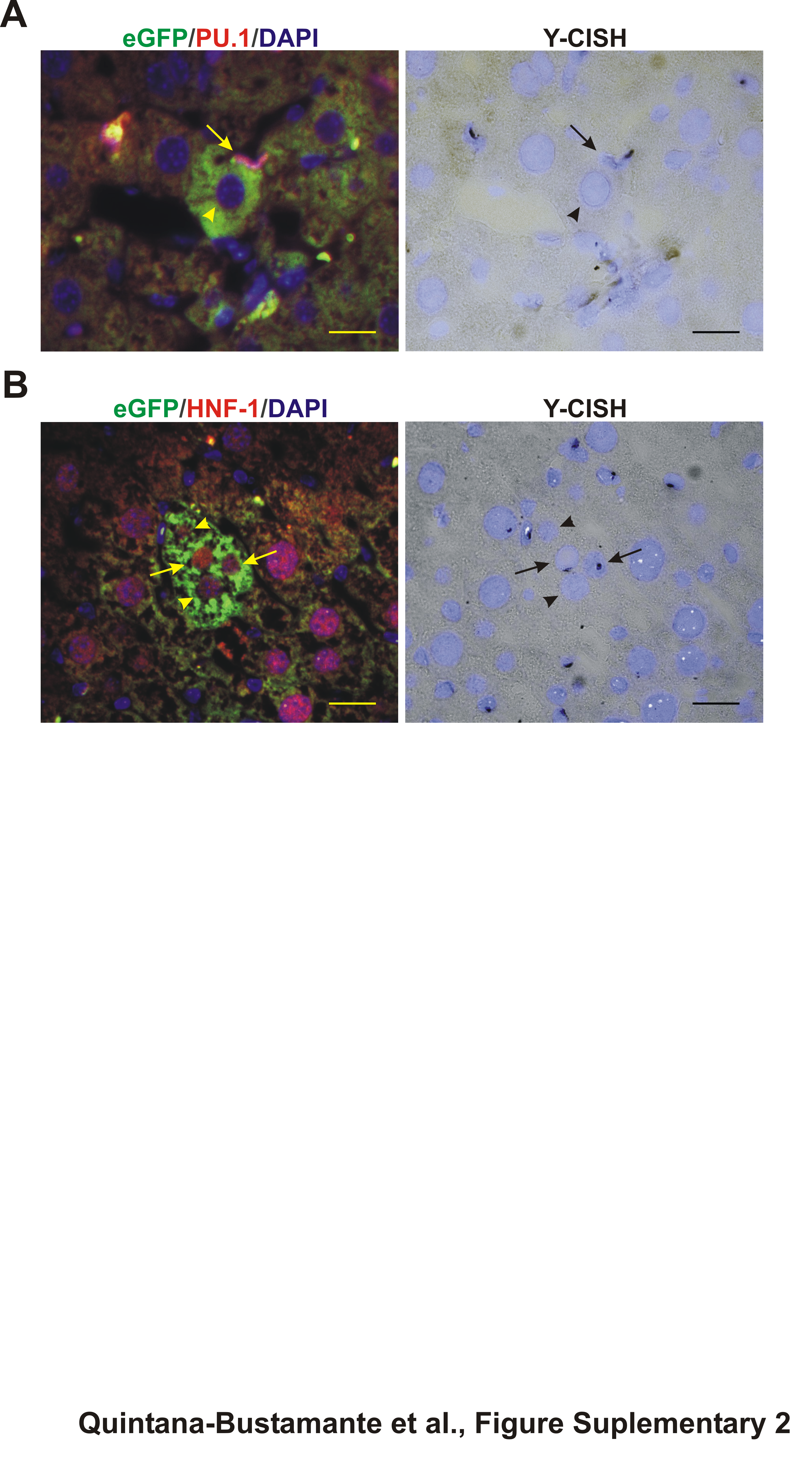

Supplement: Figure S2 — Identification of PU.1 and HNF-1A in BMDH nuclei. Additional examples of identification of PU.1 and HNF-1A in BMDH by immunofluorescence and Y-CISH. A. PU.1 analysis in the nuclei of BMDH originating from endogenous hepatocytes (arrowhead) or BM cells (arrows). B. Presence of HNF-1A in a multinucleated BMDH originating from endogenous hepatocytes (arrowhead) or BM cells (arrows). 20 µm scale bars are shown. (TIF) [file pone.0033945.s002.tif]

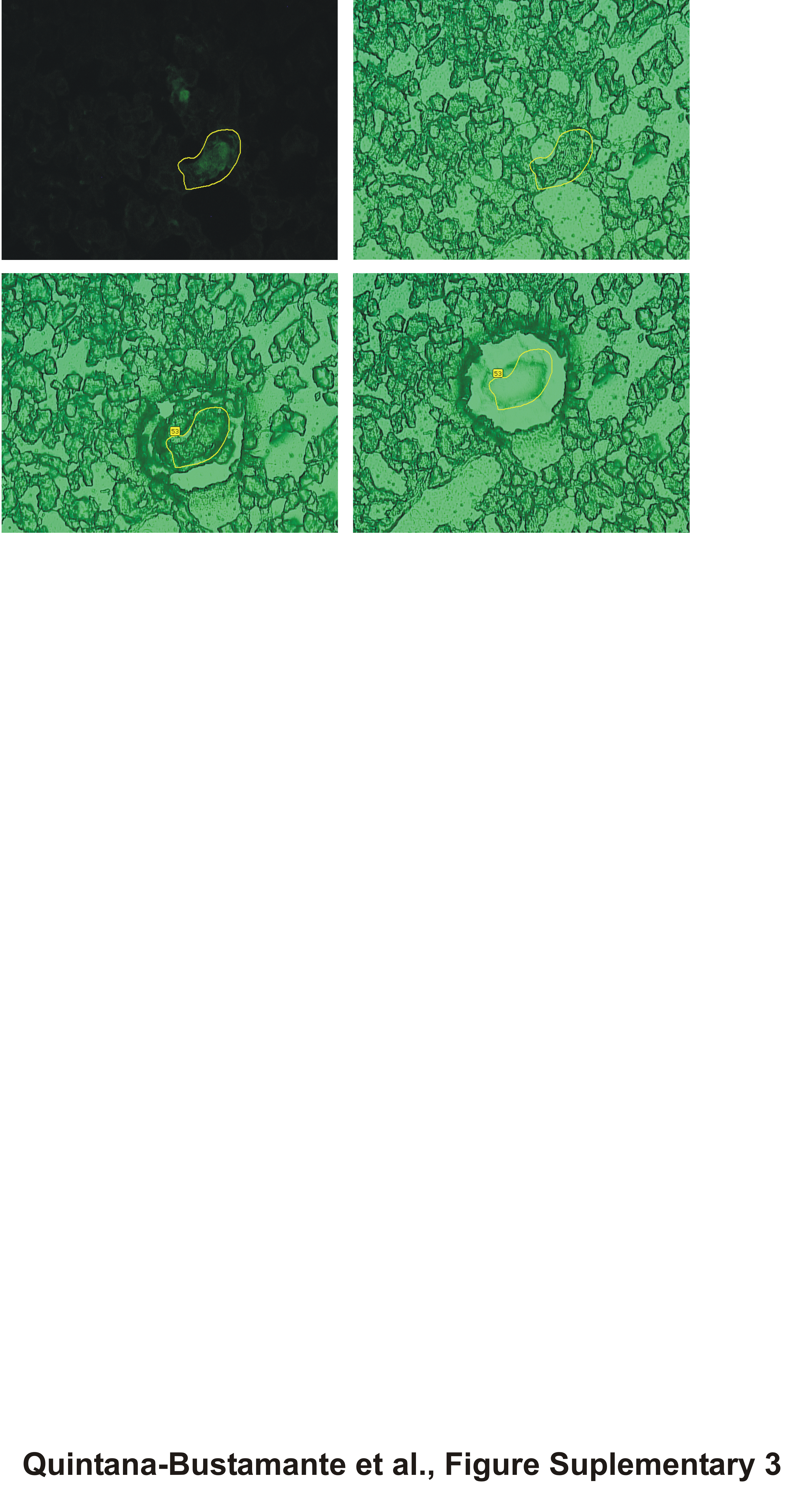

Supplement: Figure S3 — Laser capture procedure used to obtain isolated BMDH for further molecular analyses. BMDH were selected according to a clear hepatocyte like morphology (A) and their eGFP expression (B). C. To ensure that only the selected cell is captured, the tissue surrounding the cell of interest is burned out with the laser beam. D. The selected cell is catapulted to an Eppendorf tube cap for further RNA extraction. (TIF) [file pone.0033945.s003.tif]

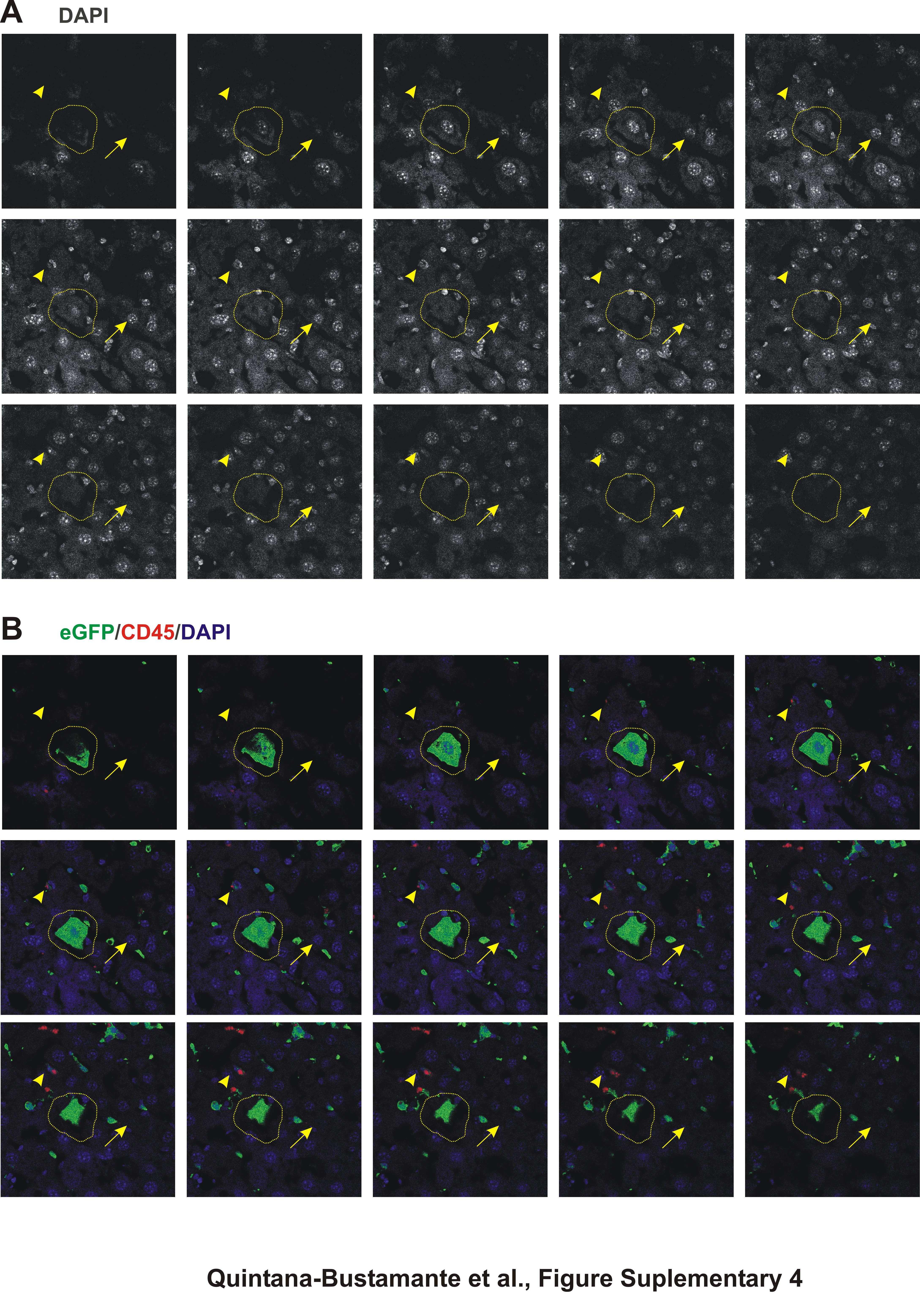

Supplement: Figure S4 — Z-stack confocal analysis. DAPI staining pattern of different nuclei (A) and identification of eGFP (green), CD45 (red) and DAPI (blue) staining (B) along Z-axis is represented as a serial 0.25 µm frames separate each 1 µm. BMDH (dotted line), hematopoietic (arrowhead) and hepatocyte (arrow) nuclei are shown. (TIF) [file pone.0033945.s004.tif]

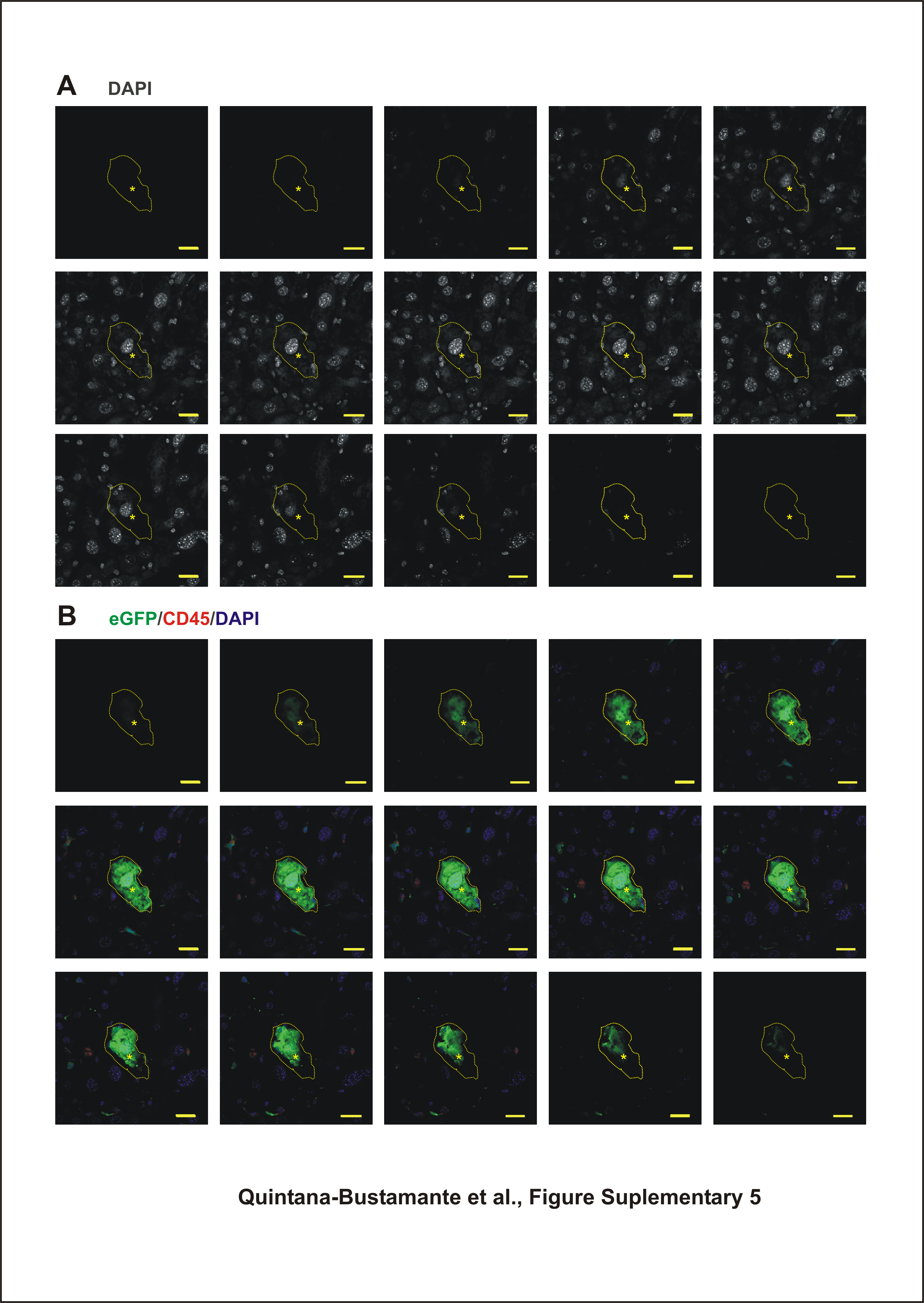

Supplement: Figure S5 — Z-stack confocal analysis of a BMDH with a Type I nucleus. DAPI staining pattern of different nuclei (A) and identification of eGFP (green), CD45 (red) and DAPI (blue) staining (B) along Z-axis is represented as a serial 0.25 µm frames separate each 1 µm. BMDH (dotted line) and hepatocyte (Type I, asterisk) nuclei are shown. (TIF) [file pone.0033945.s005.tif]

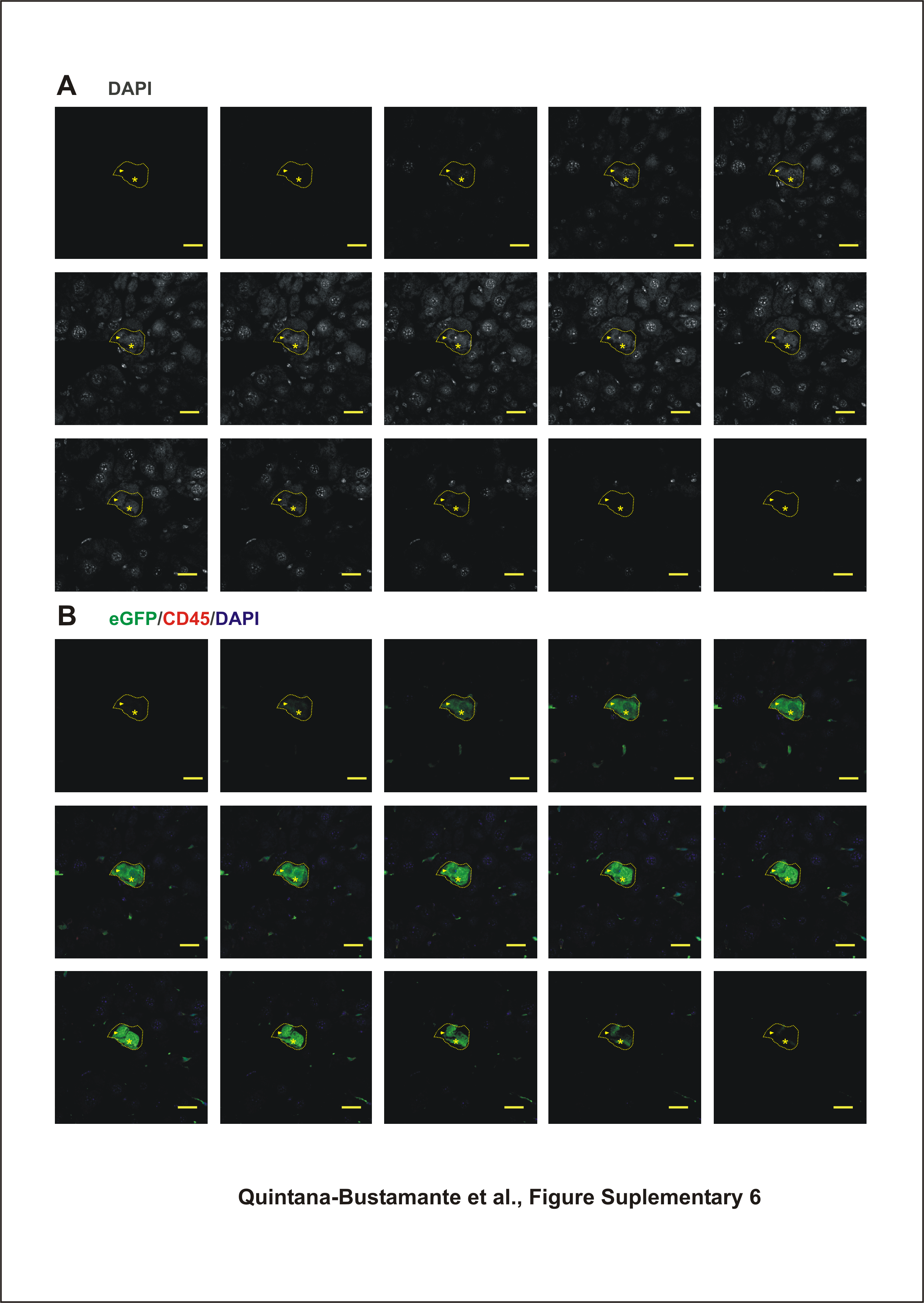

Supplement: Figure S6 — Z-stack confocal analysis of a BMDH with a Type I and a Type II nuclei. DAPI staining pattern of different nuclei (A) and identification of eGFP (green), CD45 (red) and DAPI (blue) staining (B) along Z-axis is represented as a serial 0.25 µm frames separate each 1 µm. BMDH (dotted line), Type II (arrowhead) and hepatocyte (Type I, asterisk) nuclei are shown. (TIF) [file pone.0033945.s006.tif]

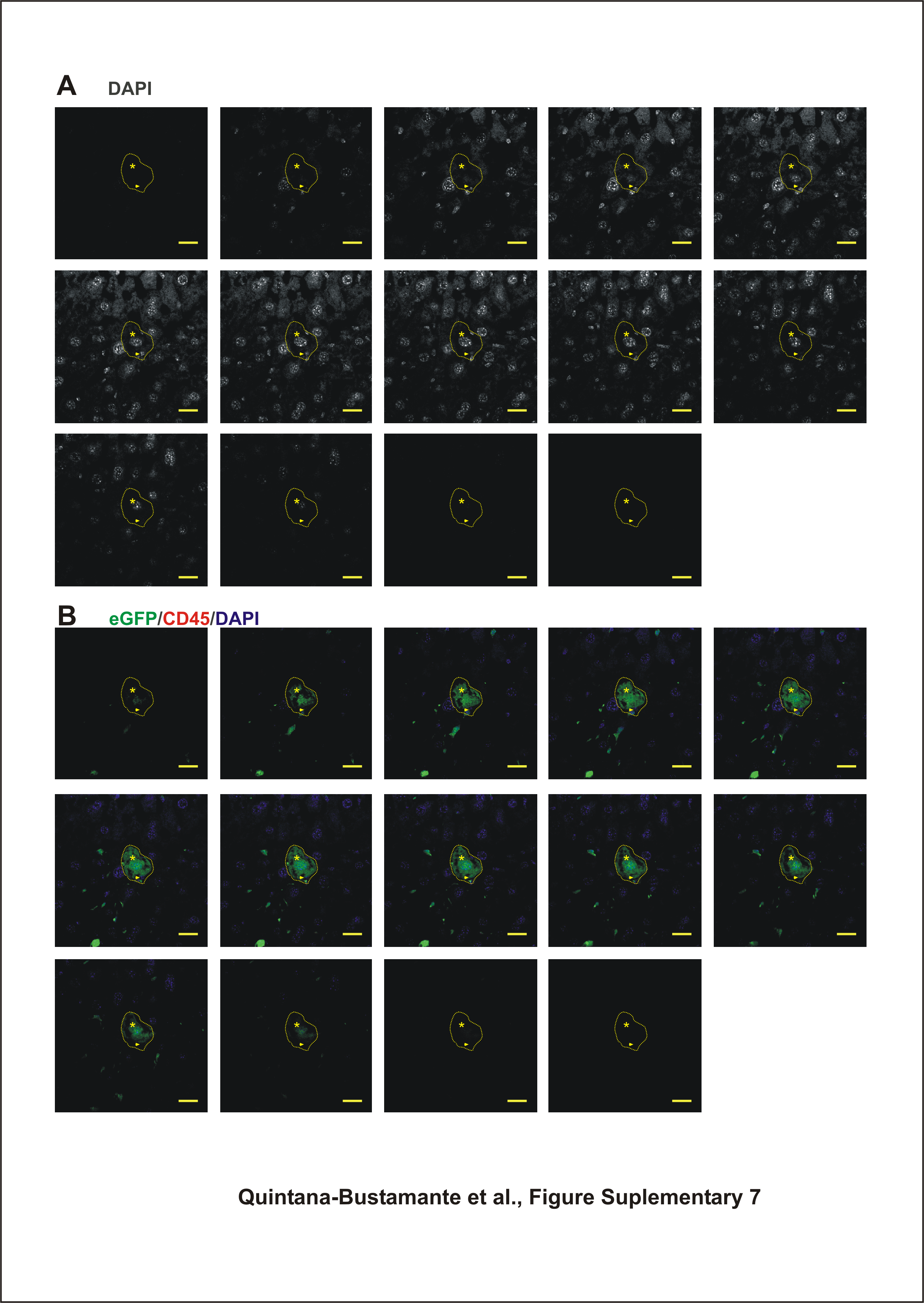

Supplement: Figure S7 — Z-stack confocal analysis of a BMDH with a Type I and a Type III nuclei. DAPI staining pattern of different nuclei (A) and identification of eGFP (green), CD45 (red) and DAPI (blue) staining (B) along Z-axis is represented as a serial 0.25 µm frames separate each 1 µm. BMDH (dotted line), Type III nucleus (arrowhead) and hepatocyte nucleus (Type I, asterisk) are shown. (TIF) [file pone.0033945.s007.tif]
